# Life with one kidney

**DOI:** 10.1007/s00467-017-3686-4

**Published:** 2017-05-29

**Authors:** Michiel F. Schreuder

**Affiliations:** Department of Pediatric Nephrology, Amalia Children’s Hospital, Radboud University Medical Center, PO Box 9101, 6500 HB Nijmegen, The Netherlands

**Keywords:** Solitary functioning kidney, Unilateral renal agenesis, Multicystic dysplastic kidney, Glomerular hyperfiltration, Childhood

## Abstract

Life with a solitary functioning kidney (SFK) may be different from that when born with two kidneys. Based on the hyperfiltration hypothesis, a SFK may lead to glomerular damage with hypertension, albuminuria and progression towards end-stage renal disease. As the prognosis of kidney donors was considered to be very good, having a SFK has been considered to be a benign condition. In contrast, our research group has demonstrated that being born with or acquiring a SFK in childhood results in renal injury before adulthood in over 50% of those affected. Most congenital cases will be detected during antenatal ultrasound screening, but up to 38% of cases of unilateral renal agenesis are missed. In about 25–50% of cases of antenatally detected SFK there will be signs of hypertrophy, which could indicate additional nephron formation and is associated with a somewhat reduced risk of renal injury. Additional renal and extrarenal anomalies are frequently detected and may denote a genetic cause for the SFK, even though for the majority of cases no explanation can (yet) be found. The ongoing glomerular hyperfiltration results in renal injury, for which early markers are lacking. Individuals with SFK should avoid obesity and excessive salt intake to limit additional hyperfiltration. As conditions like hypertension, albuminuria and a mildly reduced glomerular filtration rate generally do not result in specific complaints but may pose a threat to long-term health, screening for renal injury in any individual with a SFK would appear to be imperative, starting from infancy. With early treatment, secondary consequences may be diminished, thereby providing the optimal life for anyone born with a SFK.

## Introduction

Life with a solitary functioning kidney (SFK) has been considered by most physicians to be similar to living with two kidneys [1], a perception generally based on the excellent prognosis of kidney donors [2, 3]. Any SFK must perform the renal work normally undertaken by two kidneys. This compensatory adaptation is based on hyperfiltration of the nephrons [4, 5], the functioning units of the kidney, which is present but considered to be a harmless response in a SFK to the reduction in functional nephron number. The last decade, our research group has performed the KIMONO study (KIdney of MONofunctional Origin) with the aim to study the development of renal injury in children with different origins of SFK. With this study we have demonstrated that a congenital SFK may lead to renal injury with hypertension and albuminuria, as well as to renal functional decline that may end in end-stage renal disease (ESRD) [6–9]. The aim of this review is to discuss various issues that may be encountered in the life of an individual born with one kidney.

## Fetal life

### Ultrasound screening

The introduction of routine ultrasound assessment of the neonate has led to the recognition that a congenital SFK is more common than previously thought. The main two abnormalities resulting in a SFK are unilateral multicystic dysplastic kidney (MCDK), with an estimated incidence of one in approximately 4300 births, [10] and unilateral renal agenesis (URA), estimated to occur in one in approximately 2000 births [11]. Combining these data, a SFK can be expected in approximately one in every 1400 births.

Two ultrasound findings may lead to the suspicion of a SFK, i.e. an empty renal fossa or the presence of a dysplastic kidney. Renal ectopia may be one reason for an empty renal fossa that does not indicate an SFK. The potential difficulty in identifying an ectopic kidney by antenatal sonography [12] is one of the indications for postnatal follow-up. Renal dysplasia is a term used for kidneys that have formed, but development has been abnormal [13]. With this maldevelopment, renal dysplasia may be expected to result in fewer nephrons being formed and therefore for the kidney to be smaller on the (prenatal) ultrasound, with an increased echogenicity (renal hypodysplasia). While this expectation holds true for a limited number of cases, most dysplastic kidneys present as large, bright kidneys on ultrasound [14]. Cysts may also be present, leading to the condition being referred to as cystic dysplastic kidneys. When multiple cysts are present in a completely abnormal dysplastic kidney, the abnormality is referred to as a MCDK.

In individuals with normal ultrasound findings, several scenarios may still lead to a SFK. It should also be noted here that the adrenal may be mistaken for the kidney on the ultrasound scan and fill up the renal fossa [12]. Later during gestation, the retroperitoneal colon can also suggest the presence of a kidney. In a large European evaluation, only 62% of URA were detected by antenatal ultrasonic screening, illustrating these caveats [15].

A (dysplastic) kidney may regress during fetal life and therefore be present on antenatal ultrasound scans but absent from such tests later in life. This is a well-known phenomenon for MCDKs, which have been described to completely involute as early as 29 weeks of gestation [16]. The disappearance is the result of regression of the dysplastic kidney with reabsorption of the cysts, which may result in a remnant that cannot be identified by ultrasonography (in approximately 5% of MCDKs before birth [10]). However, this remnant may still be present and identified by retroperitoneoscopy [17].

When the kidney is indeed absent on one side, this is generally referred to as an URA, even though renal aplasia has been suggested to be the most frequent cause of non-formation of a kidney [18].

### Hypertrophy of the SFK

The remaining kidney may show hypertrophic growth in utero, as is described in 24–46% of MCDK cases [10]. This is most likely explained by compensatory nephron formation that can take place in the SFK during nephrogenesis. As nephrogenesis ceases around the 36th week of gestation [19], additional nephrons can only be formed in cases of congenital SFK and not in an SFK that is acquired after birth (for instance due to a nephrectomy in infancy or childhood). In various animal models of renal mass reduction during nephrogenesis, the number of nephrons in the SFK increases by 4 to 50% [9]. Although it is currently not possible to ascertain nephron numbers in living subjects, an increase in both kidney weight (+80%) and nephron number (+56%) has been reported in a single human case of congenital SFK [20]. Unfortunately, renal size in adulthood is not helpful in estimating nephron number as only about 10% of the large inter-individual variation in nephron numbers is explained by the variation in kidney size [21]. Determination of nephron numbers in vivo awaits further technological development, even though progress is being made in animal models [22].

The pathways that underlie renal hypertrophy are not yet fully understood, nor is the trigger for why hypertrophic growth is initiated. As stated previously “why should a fetus with adequate placental clearance of metabolic wastes need increased renal size?” [23]. Most animal studies of unilateral nephrectomy have shown contralateral hypertrophy, illustrating the ubiquitous compensation. The results of some studies have implied a role of the renal nerves in compensatory hypertrophy [24] while, conversely, in other studies renal denervation failed to prevent the compensatory growth of the kidney in the young [25] and adult rat [26]. These results indicate that renal innervation may have a specific role in nephrogenesis rather than nephron enlargement. Previous studies have indeed demonstrated that the renal nerves play an important role in renal development [27].

In addition to the renal nerves, a number of other pathways have been identified. Chen et al. studied normal kidney growth and renal hypertrophy following uninephrectomy and showed that uninephrectomy results in an immediate increase in blood flow to the SFK [28]. The increased renal blood flow results in a higher amino acid content in the proximal tubules, presumably due to the absorption of filtered amino acids, which subsequently leads to a direct activation of mTORC1 (mammalian target of rapamycin complex 1) signaling [28]. Increased mTORC1 activity in the remaining kidney not only stimulates ribosome biogenesis and protein synthesis but also modulates a range of cellular activities that are essential for cell growth and renal hypertrophy [29].

### Additional abnormalities in individuals with an SFK

In an individual with a congenital anomaly of the kidney and urinary tract (CAKUT), such as a SFK, additional abnormalities of the urinary tract may be anticipated. For example, vesicoureteral reflux (VUR) is frequently found (24% of individuals with URA [11] and 18–20% of those with MCDK [10]). In our KIMONO cohort, 26% of children with a congenital SFK did have an additional CAKUT [8].

The most frequently found abnormality in utero is a dilatation of the renal pelvis. The amount of urine produced is based on the fluid intake and not on the number of kidneys or nephrons present. Therefore, a SFK will have to deal with a doubling of the urine flow. With an increased flow, the pressure in the urinary tract, especially in the renal pelvis, will increase, based on Pouseuille’s law [30], and is also seen in individuals with a relative polyuria (such as nephrogenic diabetes insipidus [31]). It may therefore be anticipated that the number of children with a SFK identified as having antenatal hydronephrosis will be higher than in those without a SFK.

Extra-renal abnormalities are identified in 15–30% of individuals with a SFK [10, 11]. Some of these associated abnormalities fall within the context of a syndrome, such as the nail-patella syndrome or the branchio-oto-renal syndrome. In such cases, genetic mutations are frequently identified (for an overview of the genetics in both syndromic and isolated forms of SFK, the reader is referred to [9]). Isolated forms of SFK have been proven to be more difficult to link to a specific mutation, but candidate genes are increasingly being identified [32].

## Childhood

### Postnatal analysis

In newborns with antenatal suspicion of CAKUT, postnatal analysis will minimally consist of an ultrasound, and renal scintigraphy may also be performed to confirm the absence of an ectopic functioning kidney.

The debate on the need for a voiding cystourethrogram (VCUG) in all newborns with an SFK is similar to that regarding the indications after a urinary tract infection (UTI): the question remains of whether the detection of low-grade VUR adds value to the treatment of the patient [33]. The absence of significant pelvic dilatation is frequently used as a marker for the absence of high-grade VUR. Unfortunately, the predictive value of ultrasound findings for VUR is quite poor [34]. In individuals with SFK, the presence of VUR is associated with a higher risk of renal injury [8] and, therefore, the need to perform a VCUG may be higher than the need in the general population as it may assist in counseling of the family.

### Postnatal function

In the normal kidney, the glomerular filtration rate (GFR) increases rapidly after birth to take on the role of maintaining the body fluid balance and clearing the body of waste products [35]. A similar accelerated increase in GFR has been observed in young children with a SFK [36]. In our KIMONO paediatric cohort, we performed generalized estimated equation analyses on GFR data, which showed that GFR reaches its peak at a later time in children with a SFK, around the age of 8–10 years [7]. Irrespective of the timing, GFR in individuals with a congenital SFK does reach a normal two-kidney level of around 100 ml/min/1.73m^2^ on average, which is higher than the GFR attained after donor nephrectomy (approx. 75 ml/min/1.73m^2^) [37]. The approximately 100% increase in GFR from the SFK in congenital cases versus the approximately 50% increase after nephrectomy in adulthood points to a difference in compensatory hyperfiltration. This has been quantified on single-nephron GFR (SNGFR) levels in a rat study [38]. After nephrectomy in adulthood, SNGFR increased by 47% on average; in contrast, in rats who underwent nephrectomy early in infancy SNGFR was increased by 115% [38]. These numbers are remarkably similar to the increase in GFR as described above. All in all, these studies illustrate that at least the degree of compensatory hyperfiltration is different between acquiring an SFK early versus later in life.

**Fig. 1:**
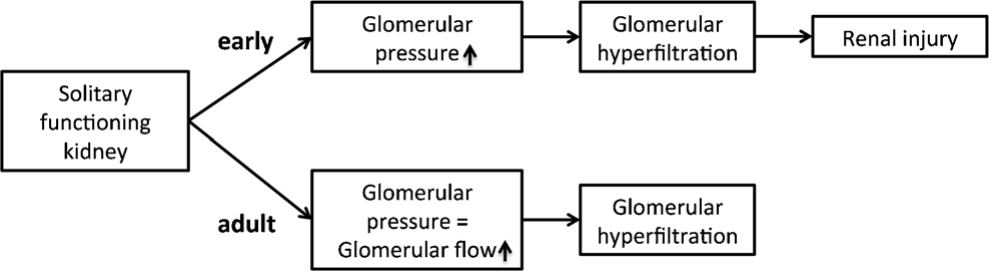
Hypothesis: Differences in pathophysiology leading to glomerular hyperfiltration explain the difference in the incidence of renal injury between nephrectomy in adulthood and a solitary functioning kidney from birth or childhood

It is currently unknown what drives this difference in glomerular hyperfiltration, but the pathways that underlie the glomerular hyperfiltration are likely to differ depending on the age at which renal mass reduction occurs. Lenihan et al. studied glomerular haemodynamics in adult kidney donors and demonstrated glomerular hyperfiltration but without a clear increase in glomerular pressure [39]. Similarly, an animal study of 5/6 nephrectomy in adult rats showed that the pressure in glomerular capillaries was only slightly (approx. 10%) increased [40]. In contrast, nephrectomy in newborn guinea pigs, in which nephrogenesis ceases prior to birth, resulted in a 30% increase in glomerular filtration pressure [41]. As glomerular haemodynamics play an important role in the development of renal injury [5], these results illustrate a clear distinction in the consequences of renal mass reduction between adulthood and early in life (Fig. 1). These differences in outcomes suggest that loss of renal mass early in life incurs a greater risk of renal and cardiovascular disease than loss of a kidney later in life [42].

Several—interrelated—pathways have been identified to be likely involved in the compensatory glomerular hyperfiltration. These are the renin–angiotensin system (RAS) [5], the nitric oxide (NO) system [43] and the vasopressin system [44].

The RAS is stimulated in many renal diseases, including diabetes-induced glomerular hyperfiltration. Indeed, inhibition of the RAS is the mainstay of early intervention in diabetic nephropathy, which is the most common cause of ESRD in adults [5]. In children with renal failure, RAS inhibition has been shown to provide a substantial benefit by slowing the decline in renal function [45]. NO plays major roles in the regulation of glomerular haemodynamics and sodium reabsorption [46]. Recently, a marked disruption of the renal NO system in sheep with a congenital SFK was observed [47]. NO also has important functions in the regulation of renal blood flow, GFR and sodium reabsorption [46], and an inhibited NO system has been shown to stimulate the RAS [48]. There is also a clear interrelation between the RAS and vasopressin, as vasopressin antagonists are able to potentiate the renoprotective properties of RAS inhibitors [49]. However, only in recent years has the role of vasopressin in the pathways that lead up to kidney disease been investigated further [44]. Moreover, vasopressin induces an endothelium-dependent relaxation of blood vessels that is NO driven [50], illustrating that the RAS, NO and vasopressin systems are interconnected. To understand their specific roles in glomerular hyperfiltration in the SFK, researchers need to focus additionally on the potential to intervene in these systems, as well as the differences in the contribution of these systems between congenital and acquired SFK.

The pathways that link glomerular hyperfiltration to glomerular damage in the SFK have not been extensively studied. In part, the increased glomerular pressure may account for this via a mechanism that is similar to diabetic- or obesity-induced hyperfiltration. It has been shown that a SFK does expose the podocytes to an increased flow of ultrafiltrate in Bowman’s space, which results in an increased fluid flow shear stress [51]. This is accompanied by an activation of the cyclooxygenase2-prostaglandin axis, which could, at least in part, be prevented by indomethacin [52]. Podocyte injury is well accepted as being a step towards glomerulosclerosis [53].

### Hyperfiltration injury

Using a systematic review, our research group studied the occurrence of hyperfiltration injury in 43 published cohorts of URA [11]. Analysis of data on 2684 patients identified hypertension in 16% of the patients, albuminuria in 21% and a reduced GFR in 10%. In order to further study the consequences of the higher degree of hyperfiltration, we designed the KIMONO study [7]. The KIMONO cohort is the largest cohort of children with a congenital SFK and acquired SFK in early childhood, comprising more than 400 children. Data from the KIMONO study have revealed that by the age of 10 years, one in three enrolled children, with either form of SFK, has indicators of renal injury and that more than 50% of the children born with an SFK develop signs of renal injury by the age of 18 years [7, 8].

In the KIMONO study renal injury was defined on the basis of the hyperfiltration hypothesis [4, 5] and consists of hypertension, albuminuria, the use of antihypertensive/antiproteinuric drugs and/or a reduced GFR [8]. Estimation of GFR during hyperfiltration may demonstrate the well-known limitations of creatinine, while cystatin C as a marker for early renal functional demise has indeed been shown to be superior to creatinine in SFK patients [54]. However, all of these findings may be quite late in detecting renal injury, as is well recognized in hyperfiltration injury in diabetic kidney disease [55]. Unfortunately, there is a lack of sensitive early diagnostic markers that are needed to intervene at an earlier time point than after the onset of irreversible glomerular damage.

The KIMONO study also revealed that a SFK due to acquired kidney loss during childhood, as compared to a congenital loss, is associated with an even greater incidence of renal injury and hypertension [7]. This is probably explained by the difference in age at follow-up, but other explanations are possible as well. Fitting with the hypothesis that additional nephron formation in congenital SFK results in a degree of protection from renal injury, the study identified a relationship between small kidney length and renal dysfunction and arterial pressure in children with a SFK, with an acquired SFK being associated with a lesser degree of compensatory renal growth [7, 8]. Therefore, one of the key differences between a congenital and acquired SFK is the degree of nephron deficit; the deficit being more severe in acquired SFK as it occurs after the completion of nephrogenesis.

### Sports

In their study of sport-related kidney injury among high school athletes in the USA, Grinsell found that in 4.4 million games or sport practices, there were 23,666 physical injuries reported of which 18 were injuries to the kidney [56]. None of these injuries to the kidney required surgery or resulted in the loss of a kidney, which does not support any limitations in sport participation in SFK individuals [57]. It is well known that physical activity is healthy and reduces the risk for several chronic diseases [58]. As individuals with a SFK have an increased risk of renal injury, sport participation should be stimulated rather than limited. Indeed, a recent study showed increased cardiorespiratory fitness after exercise training in children with a congenital SFK [59], as expected. In general, sport participation has been shown to reduce glomerular hyperfiltration [60], a finding which hopefully removes any remaining barrier in supporting a healthy and active lifestyle in individuals with a SFK even before they reach a state of chronic kidney disease (CKD), at which time exercise is again advised [61].

### Diet

Based on the original studies by the group of Brenner, a reduction of protein intake may be the cornerstone to prevent glomerular damage in the long run [62]. Recent studies also show the influence of protein intake early in life on glomerular dynamics. In a rat model of low birth weight with subsequent nephron reduction and thus glomerular hyperfiltration, an increase in protein intake resulted in glomerular hypertrophy, proteinuria and glomerulosclerosis [63].

Another example of glomerular hyperfiltration is found in individuals with marked obesity [64]. One of the pathways involved in the increase in blood pressure is the increased tubular reabsorption of sodium. Sodium restriction, together with weight loss, forms the cornerstone of therapy for obesity-associated hypertension [65]. Similar strategies are applicable to obese and overweight children and adolescents as well [66]. Whether these strategies apply to children with a SFK remain to be determined, as such studies have not been undertaken, but this is highly likely to be the case. Salt reduction in general may assist in preventing cardiovascular disease [67], and this holds especially true in vulnerable individuals such as children with kidney diseases [68, 69], making it likely that children with a SFK may benefit from a reduced salt intake. Whether this reduction in sodium intake should start from birth, which has been shown to have a long-term lowering effect on blood pressure [70], is open to debate.

## Adult life

### Renal function and injury

Data on the long-term consequences of being born with a SFK are limited. As a consequence, data from adults that underwent donor nephrectomy have been frequently used. Such studies have generally shown no (renal) disadvantage over age- and sex-matched controls, even 40 years after nephrectomy [71]. Unfortunately, the controls that were used in such studies came from the general population, whereas the donors came from a much lower-risk population as they needed to be (extremely) healthy to be allowed to donate a kidney. Data from more recent studies that have used different control groups, such as healthy non-donors that are matched to the donors, show that there is indeed a significant, albeit small, increased risk of ESRD in the donor cohort [72].

Most of the information currently available on the renal outcome of a congenital SFK derives from the study of Sanna-Cherchi et al., who demonstrated that 20–40% of patients with a SFK require renal replacement therapy by the age of 30 years [73]. It may be argued that this study represents a selected cohort, as the cohort was not formed on the basis of antenatal detection of a SFK and may therefore be biased towards a worse prognosis. However, the results clearly show that being born with a SFK is not a benign condition per se. In a cohort from China of 48 SFK adults at a mean age of 36.7 years, 38.5% had a GFR of <60 ml/min/1.73 m^2^ and two had started dialysis [74]. In this study, individuals with a SFK length of <12.0 cm were found to have a 5.5-fold higher risk for a reduced GFR [74]. These results are in line with our KIMONO data that also identified renal size to be associated with the risk to develop renal injury [8].

Unfortunately, to date there have been no large, longitudinal studies beyond adolescence in individuals with a congenital SFK. The cohorts discussed above and others from the literature [75, 76] are therefore likely to suffer from selection bias, which makes it difficult to realistically predict future health in individuals with a congenital SFK. With the availability of routine ultrasound screening in pregnancy, cohorts can be constructed that encompass the entire range of individuals with a congenital SFK, including the healthy ones. Such prenatally defined cohorts will not only enable the natural course of a SFK to be studied, but they will also allow for the identification of prognostic markers that may assist in defining a personalized follow-up scheme. Based on data from the KIMONO cohort, our research group has included only the presence or absence of additional CAKUT and of renal injury in our recommendation for clinical follow-up (Table 1) [9]. As a high rate of masked hypertension has been observed [77], it may be wise to not solely rely on office blood pressure reading but to also include ambulatory blood pressure measurements in the follow-up of SFK children.

**Table 1:** Opinion-based recommendation for clinical follow-up of children with a solitary functioning kidney

Clinical parameter	No renal injury		Renal injury: GFR <60 ml/min/1.73 m^2^, (use of medication for) hypertension and/or proteinuria
	CAKUT−	CAKUT+	
Blood pressure^a^	One time per year	Two times per year	Two to four times per year
Albuminuria	One time per year	Two times per year	Two to four times per year
Serum creatinine/eGFR	Every 5 years	Every 5 years	Two to four times per year
Renal ultrasound	Every 5 years	As indicated	As indicated

Reproduced from Westland et al. [9], used with permission

CAKUT, Congenital anomalies of the kidney and urinary tract; GFR, glomerular filtration rate; eGFR, estimated GFR

^a^Based on the high rate of masked hypertension [77], ambulatory blood pressure measurement should be considered

### First hit–second hit hypothesis

According to the “first hit–second hit” hypothesis, a low nephron number may influence the presentation and course of any additional kidney disease, thereby altering its prognosis. This has been shown for several kidney diseases in patients with low birth weight and subsequent low nephron numbers [78]. As a SFK is another example of a congenital nephron deficit, the same may apply for individuals with a SFK. A review of eight published cases of patients with autosomal dominant polycystic kidney disease in the setting of a SFK did not allow for a conclusion to be drawn on the rate of disease progression in such patients [79].

McDonald et al. studied the risk of contrast-induced acute kidney injury in a cohort of 264 SFK patients, of whom at least 80% had a nephrectomy in adulthood [80]. Their analysis of the risk with a propensity score-matched control group did not show an increased risk of acute kidney injury in the SFK cohort. Of interest are the data on the demographics of their SFK cohort, which shows hypertension in 65% and CKD in 39%. This was significantly higher than the control cohort with 47% and 10%, respectively [80], highlighting the potential health issues in any individual with an SFK.

### Pregnancy

Being pregnant results in several renal adaptations, including glomerular hyperfiltration [81]. In the case of limited renal function, pregnancy may result in such an increased that the result is kidney injury. In addition, kidney disease, even mild, may increase maternal as well as fetal risk [82]. These risks have been studied in kidney donors as well, with the results showing that gestational hypertension and preeclampsia are 2.4- to 2.5-fold more common in the kidney donor group [83]. When pre- with post-donation pregnancies are compared, the risk for the composite endpoint of gestational diabetes, hypertension and preeclampsia is more than fivefold increased in the latter [84], illustrating the risks for a potential kidney donor with a pregnancy wish. Whether this holds true for women with a SFK remains to be determined, as such data are not yet available.

An additional risk for the offspring may be found in the increased risks for family members of an individual with a SFK to have a urogenital anomaly [85]. In part, this is based on the genetic abnormalities that are found in individuals with a SFK which can be passed on to the next generation. Whether the intrauterine environment of a woman with a SFK is different, and therefore results in an increased chance of CAKUT, is unknown. Animal studies have shown that nephrectomy in early pregnancy increases nephron numbers in the offspring [86], illustrating that alterations in the fetal development may be anticipated.

### Sex differences

The issues regarding pregnancy could suggest that men with a SFK are better off than women with regards to the risks for any kidney-associated problem later in life. In general, the opposite is true—with a better outcome in females with CKD [87]. However, no differences in terms of renal injury risks between the sexes were found in the KIMONO cohort [88]. As the difference between the sexes really comes into play from puberty onwards, as sex hormones may have the greatest impact after puberty, and the KIMONO cohort was relatively young [mean age 9.0 (standard deviation 6.0) years] [8]), the effect of gender may have been studied too early in life in the KIMONO cohort. Indeed, several studies on the effect of gender on compensatory mechanisms and long-term sequelae in SFK have shown that male animals show a higher degree of hyperfiltration [89], have a higher blood pressure [90] and higher glomerular pressure and more hypertrophy, and that testosterone is indeed the driving force in this difference [91].

### SFK: All bad?

Based on the data presented, it may be concluded that nothing good is expected when born with a SFK. The data indeed do show that there is an increased risk of renal injury, and many individuals with a SFK from childhood or even before birth do have an indication to be subjected to life-long follow-up. Indeed, similar to diabetes, the longer the hyperfiltration is continued, the higher the chances that renal injury develops. However, based on the results of the KIMONO study, it cannot be stated that all individuals with a SFK will develop renal injury. Unfortunately, markers to differentiate between the groups with increased risk and the group with a standard risk are currently lacking, even though the standard risk may also be different when an additional stressor (either kidney disease or pregnancy) is present.

In contrast, there is a study that points towards a benefit of having an SFK—a study in dogs showed that after uninephrectomy the remaining kidney is more resistant to ischaemia [92].

## Conclusion

Being born with a SFK is not a benign condition per se. The compensatory hyperfiltration is associated with increasing renal injury, starting early in childhood. At several times in life, consequences may present themselves to an individual with a SFK. As conditions like hypertension, albuminuria and a mildly reduced GFR generally do not result in specific complaints but may pose a threat to long-term health, screening for renal injury in any individual with a SFK seems imperative, starting from infancy. With early treatment, secondary consequences may be diminished, thereby providing the optimal life for anyone born with a SFK.

## Key summary points

- Being born with or acquiring in childhood a SFK results in renal injury in over 50% of those affected before adulthood.
- Renal injury in a SFK is in line with Brenner’s hyperfiltration hypothesis: extra attention to prevent other causes of glomerular hyperfiltration, such as obesity, is needed.
- Follow-up in all patients with a solitary functioning kidney is imperative

## Multiple Choice Questions (answers are provided following the reference list)

1. The incidence of SFK is estimated to be:
  - One in approximately 500
  - One in approximately 1400
  - One in approximately 2000
  - One in approximately 4300
2. The most common etiology of SFK is:
  - Genetic
  - Associated with gestational diabetes
  - Drug use during gestation
  - Unknown
3. The sequence of events in the hyperfiltration hypothesis is:
  - Systemic hypertension, glomerular hypertension, GFR increase, albuminuria
  - Systemic hypertension, glomerular hyperfiltration, albuminuria, GFR decline
  - Glomerular hyperfiltration, glomerular hypertension, systemic hypertension, GFR decline
  - Glomerular hyperfiltration, albuminuria, GFR increase, systemic hypertension
4. Follow-up in patients with a SFK:
  - Is only indicated in specific circumstances, such as a GFR <60 ml/min/1.73 m^2^
  - Is indicated in all children with a SFK
  - Is indicated in all patients with a SFK, with a reduced intensity of follow-up after 10 years
  - Is indicated in all patients with a SFK

5. Which of the following factors is NOT associated with an increased risk of renal injury in congenital SFK:
  - Male sex
  - Increasing age
  - Additional anomalies in the SFK or urinary tract renal hypertrophy
